# Inappropriate self-medication among adolescents and its association with lower medication literacy and substance use

**DOI:** 10.1371/journal.pone.0189199

**Published:** 2017-12-14

**Authors:** Chun-Hsien Lee, Fong-Ching Chang, Sheng-Der Hsu, Hsueh-Yun Chi, Li-Jung Huang, Ming-Kung Yeh

**Affiliations:** 1 Department of Health Promotion and Health Education, National Taiwan Normal University, Taipei, Taiwan, ROC; 2 Department of Pharmacy Practice, Tri-Service General Hospital, National Defense Medical Center, Taipei, Taiwan, ROC; 3 Division of General Surgery, Department of Surgery, Tri-Service General Hospital, National Defense Medical Center, Taipei, Taiwan, ROC; 4 Department of Health Developing and Marketing, Kainan University, Taoyuan, Taiwan, ROC; 5 Chia Nan University of Pharmacy and Science, Tainan, Taiwan, ROC; 6 Ministry of Health and Welfare, Taipei, Taiwan, ROC; The Chinese University of Hong Kong, HONG KONG

## Abstract

**Background:**

While self-medication is common, inappropriate self-medication has potential risks. This study assesses inappropriate self-medication among adolescents and examines the relationships among medication literacy, substance use, and inappropriate self-medication.

**Method:**

In 2016, a national representative sample of 6,226 students from 99 primary, middle, and high schools completed an online self-administered questionnaire. Multiple logistic regression analysis was used to examine factors related to inappropriate self-medication.

**Results:**

The prevalence of self-medication in the past year among the adolescents surveyed was 45.8%, and the most frequently reported drugs for self-medication included nonsteroidal anti-inflammatory drugs or pain relievers (prevalence = 31.1%), cold or cough medicines (prevalence = 21.6%), analgesics (prevalence = 19.3%), and antacids (prevalence = 17.3%). Of the participants who practiced self-medication, the prevalence of inappropriate self-medication behaviors included not reading drug labels or instructions (10.1%), using excessive dosages (21.6%), and using prescription and nonprescription medicine simultaneously without advice from a health provider (polypharmacy) (30.3%). The results of multiple logistic regression analysis showed that after controlling for school level, gender, and chronic diseases, the participants with lower medication knowledge, lower self-efficacy, lower medication literacy, and who consumed tobacco or alcohol were more likely to engage in inappropriate self-medication.

**Conclusion:**

Lower medication literacy and substance use were associated with inappropriate self-medication among adolescents.

## Introduction

Self-medication (SM) is commonly defined as using drugs to self-treat a common health problem without a physician’s advice [1]. The World Health Organization advocates self-care and responsible SM [2]. SM may have some benefits if individuals use medicine appropriately, such as empowering individuals to take care of themselves and be responsible for their health and further reducing healthcare costs [3]; however, responsible SM does not imply an absence of risk. Several studies have reported that over-the-counter (OTC) use is associated with adverse health reactions [4] and fatalities [5, 6]. SM carries risks if individuals use medicine inappropriately. The risks of SM include incorrect self-diagnosis, delay in seeking medical advice, use of excessive dosages, prolonged drug-use duration, side effects, drug interactions, polypharmacy, and drug abuse [3, 7].

The United States National Institutes of Health [8] noted that individuals are not always responsible regarding how they practice SM and often do not have adequate ability to deal with the symptoms caused by their SM. Inappropriate nonprescription use is often associated with limited information and low knowledge of medication use [9]. Delays in treatment occur because of incorrect self-diagnosis [10]. Review studies have found that the majority of preventable adverse drug reactions are related to excessive dosage [11], whereas several negative consequences include adverse drug events and drug interactions associated with polypharmacy [12]. Another study reported that about one-third of instances of inappropriate OTC medicine use are to be classified as drug abuse [13], whereas the most abused OTC medicines include codeine-based (especially compound analgesic) medicines, cough products, sedative antihistamines, decongestants, and laxatives [14].

The prevalence of SM among adolescents in different countries varied from 2% to 92% [15]. For example, the prevalence of SM ranged from 4.7% to 11.3% (1-year prevalence) in the United States [16, 17], 17% to 39% (2-day prevalence) in Finland [18, 19], 31.6% (4-week prevalence) in Germany [20], 37.7% (1-year prevalence) in Saudi Arabia [21], 67% (1-year prevalence) in Sweden [22], 89.2% (1-year prevalence) in the United Arab Emirates [23], and 92% (1-year prevalence) in Kuwait [24]. The prevalence of SM among adolescents in developing countries was also high [15, 25]. A review study [15] reported that the most commonly used medications among adolescents for SM are analgesics, vitamins, and nutritional supplements, anti-allergic, and cold and cough medicines, whereas the most common health complaints leading to SM include headache, allergies, and fever [15]. Studies have reported that factors associated with the SM of adolescents include age, gender, familial practices, and tobacco and alcohol use [15, 26]. In addition, adolescents and adults engage in inappropriate SM behaviors such as not reading drug labels or instructions [27], taking excessive dosage, and polypharmacy [3, 7, 27–30].

The World Health Organization found that SM in individuals with lower medication knowledge may result in several potential risks, including incorrect self-diagnosis, delays in seeking medical advice, use of inadequate or excessive dosages, prolonged drug-use duration, drug interactions, lack of awareness of warnings and precautions, storage in incorrect conditions or beyond the recommended shelf-life, polypharmacy, and drug abuse [2]. For example, analgesics are a common nonprescription medicine, but most patients lack knowledge of analgesic use, which is related to inappropriate use of analgesics and excessive doses of acetaminophen [31–33].

In addition, low health literacy is also associated with inappropriate SM [34–36], and individuals with low health literacy tend to adhere less to self-care regimens [37], have more medication errors [38], have a higher risk of hospitalization [39], and undergo prolonged hospitalization [40]. Medication literacy is defined as knowledge of the appropriate use of medication and is a part of health literacy [41] that includes functional, interactive, and critical literacy [42]. Compared with health literacy, medication literacy requires more skill to practice [43]. Inappropriate medication use such as nonadherence, overdose, and misunderstanding drug labels or instructions is associated with limited health literacy in adults [36, 44, 45]. Patients with limited medication literacy tend to undergo more frequent re-hospitalization, emergency department visits, and serious adverse drug events [46]. Medication literacy can serve as a predictor of appropriate medication use [47].

Substance use by adolescents is an emerging public health problem [48], which may be exacerbated by SM. Studies have shown that drinking alcohol and using tobacco are associated with SM [49]. In addition, a study showed that frequent alcohol drinking was a risk factor of prescription medication use among adolescents [50]. Since adolescents’ risk behaviors often begin during adolescence and increase with age, the frequency of engagement in these behaviors often continues to rise into early adulthood [51]. However, limited research has been conducted on the relationship between substance use and inappropriate medication use, especially with respect to SM.

Taiwan has a convenient and inexpensive health care system: The coverage rate of the National Health Insurance reaches approximately 99% of the population and almost all hospitals collaborate with it [52]. However, low health literacy is prevalent among children and adults in Taiwan [53, 54]. The present study focused on assessing the prevalence of SM among adolescents in Taiwan and examining the factors of inappropriate SM.

## Materials and methods

### Participants

This cross-sectional study examined adolescents in Taiwan from primary (5th and 6th grades), middle (7th, 8th, and 9th grades), and high (10th, 11th, and 12th grades) schools in Taiwan and was conducted between September and November 2016. A representative sample of students was chosen using probability proportionate to size sampling. In 2016, there were 2,630 primary schools, 735 middle schools, and 506 high schools in Taiwan. Of these, 48 primary schools, 32 middle schools, and 29 high schools were randomly selected and two to three classes were chosen from each school to participate in this survey. A total of 99 out of 109 selected schools (90.8%) participated in the study. Students from 43 primary schools, 30 middle schools, and 26 high schools completed this survey. In all, 6,226 adolescents, including 3,055 females (49.1%) and 3,171 males (50.9%), completed this study’s self-administered online questionnaire, which was anonymous, at their schools. The results of a chi-square test showed that there was no significant difference in gender between this study’s sample and the Taiwan adolescent population. The Ethical Committee of National Taiwan Normal University reviewed and approved the present study. The consent forms were taken home by students to give to parents, requesting their consent to allow the children to participate in the survey. After the parental consent forms were collected, the online self-administered survey were conducted.

### Instruments

This study’s questionnaire was developed and modified according to those presented by previous studies [33, 55, 56]. Its content validity was assessed by a diverse expert panel comprising two family medicine physicians; seven pharmacists (three community pharmacists, three hospital pharmacists, and one policy developers); four public health educators; and two school teachers. The content validity index of the questionnaire was 0.96. The adolescents’ responses to the online survey and the questionnaire’s reliability were assessed by a pilot survey. The questionnaire comprised five parts: knowledge of correct medication use, self-efficacy of correct medication use, medication literacy, experience of SM and substance use during the last year, and the participants’ demographic information.

#### Knowledge of correct medication usage

The items for knowledge of correct medication usage were developed on the basis of previous studies on five core abilities for correct medication usage [33, 55, 56]. The first component (Ability I: being the master of yourself in taking medications) is the ability to balance the benefits and risks of medication to make your decision on self-care or medication use. The second component (Ability II: expressing personal conditions clearly) is the ability to clearly inform physicians or pharmacists of your personal conditions or needs for health care. The third component (Ability III: checking information on the medication package) is the ability to read and understand the information on drug labels or instructions. The fourth component (Ability IV: taking medications correctly) is the ability to adhere to the directions for the use of the medicine. The fifth component (Ability V: being open with pharmacists and physicians) is the ability to clearly ask pharmacists and physicians questions about a medicine when you need help or more information. Knowledge of correct medication use was measured using 14 statements (Cronbach’s α = 0.83). Sample items included the following: “Taking OTC cold medicine cannot prevent common colds”; “When common colds become severe, taking even more cold medicine could make me better faster”; and “Cold medicines containing active ingredients that help relieve a runny nose and sneezing may make a person sleepy.” The response option for each item included yes, no, and unknown. The adolescents obtained 1 point if they answered the item correctly, while they obtained 0 point if they answered the item incorrectly or unknown. The average knowledge sore (0–1) was calculated by total knowledge score (0–14) divided by the number of items (14). Higher average score indicated higher levels of knowledge in correct medication use.

#### Self-efficacy of correct medication usage

Self-efficacy of correct medication use refers to an adolescent’s confidence in making decisions for proper medicine use. It was measured using 14 statements (Cronbach’s α = 0.93). Sample questions included the following: “Before buying OTC medicines, I can discuss with pharmacists regarding which medicine to choose”; “Before taking OTC medicine, I can read medication use methods, dose, side effects, and warnings on the drug label”; “Before the medicine is all taken, I can keep the medication packaging and instructions.” The response options were assessed using a five-point Likert scale ranging from 1 (very unconfident) to 5 (very confident). Higher scores were equated with higher levels of confidence in correct medication use.

#### Medication literacy

The medication literacy item was developed on the basis of Nutbeam’s health literacy model, which includes three levels of health literacy: Level I is functional literacy, Level II is interactive literacy, and Level III is critical literacy [42]. Medication literacy is a part of health literacy that requires more skills to practice. We developed real-life scenarios and items to assess medication literacy. We designed a scenario of going to purchase and take medicine and created question items to assess medication literacy. In this domain, drug labels and instructions played an important role in assessing literacy. There were six questions (Cronbach’s α = 0.93) for this assessment. The participants were instructed to read the printed labels on a medication package and instructions before answering survey questions. The questions included the following: “Before you bought the medicine, did you know how a person should take this medicine from the labeling information on the medication package?” (functional literacy); “Do you know what the drug classification is of this medicine?” (functional literacy); “What is the expiration date of this medication?” (functional literacy); “When you need medication to relieve uncomfortable symptoms, how do you express your situation and needs to a pharmacist clearly?” (interactive literacy); “If your five-year-old sister has the same symptom as you, how could she use this medicine?” (critical literacy); and “If cough is your only symptom, how would you take this medicine?” (critical literacy). Each question had four response options with only one correct choice. Higher scores were equated with higher levels of medication literacy.

#### Experience of SM

The participants were asked about their experience of SM in the past year. They were asked whether they had used nonprescription medicines, what kind of health complaints they had used nonprescription medicine for, what kinds of nonprescription medicine they had used, and whether they kept the medication packaging and instructions until the medicine was all taken. The participants were also asked who provided the information for the nonprescription medicine they used. Inappropriate SM behaviors included not reading drug labels or instructions before use, using excessive dosages, and polypharmacy. They were asked whether they read the drug labels and instructions before using medication (read drug labels and instructions) in the past year, whether they had ever exceeded the recommended dose (excessive drug dosages) in the past year, and whether they had ever used prescription and nonprescription medicines simultaneously without being advised by health providers (polypharmacy) in the past year. The response option for these items was either yes or no.

#### Demographics characteristics and tobacco or alcohol use

The participants’ demographic characteristics included gender, household income, school level, chronic disease, and family members with a professional health background. Chronic disease was measured by asking the participants whether they had chronic diseases which taking medicines more than 3 months. In addition, the participants were asked whether they had smoked tobacco in the past year and whether they had consumed alcohol in the past year.

### Data analysis

Data were analyzed using SAS software version 9.3. Statistical analyses were performed using descriptive statistics, a chi-square test, and analysis of variance to test group differences. A model of multivariate logistic regression analysis was used to examine the factors related to SM and inappropriate SM. The missing values were not included in the logistic regression model. Finally, the adjusted odds ratio (OR) and 95% confidence intervals (95% CI) were calculated to indicate the relationships among the independent variables and SM and inappropriate SM.

## Results

### Demographic characteristics and substance use

The demographic characteristics and alcohol and tobacco use of the participants are given in Table 1. About 9% of the participants reported that they had consumed alcohol in the past year, whereas 4% reported that they had used tobacco in the past year. About 7% reported that they had chronic diseases, whereas 7% reported that they had a family member with a professional health background.

**Table 1:** Demographic characteristics and substance use by gender.

	Total		Female		Male		Chi-square test
	n	%	n	%	n	%	p value
**School level**							0.3581
Primary	1696	27.3	818	48.2	878	51.8	
Middle	2280	36.6	1106	48.5	1174	51.5	
High	2250	36.1	1131	50.3	1119	49.7	
**Household income**							< .0001
Poor	1152	18.5	499	43.3	653	56.7	
Middle and above	5074	81.5	2556	50.4	2518	49.6	
**Chronic disease**							0.2904
No	5821	93.5	2867	49.2	2954	50.8	
Yes	404	6.5	188	46.5	216	53.5	
**Family members with a professional health background**							0.0433
No	5816	93.4	2874	49.4	2942	50.6	
Yes	409	6.6	181	44.2	228	55.8	
**Alcohol use**							<0.001
No	5673	91.1	2846	50.2	2827	49.8	
Yes	551	8.8	208	37.7	343	62.3	
**Tobacco use**							<0.001
No	6004	96.5	3006	50.1	2998	49.9	
Yes	219	3.5	47	21.5	172	78.5	

Notes: Total N = 6226 Female n = 3055 Male n = 3171

### Medication knowledge

Overall, the medication knowledge of the participants was low (M = 0.81), and there was a statistically significant difference between males and females (Table 2). For example, 55% of the participants were unaware that people who drink alcoholic beverages or have hepatitis increase their risk of liver damage when taking pain medicine containing acetaminophen, whereas 29% had the mistaken belief that taking medicine combined with antacids would prevent harm to the stomach. In addition, 30% were unaware that the drug classifications of medications are based on risk, whereas 21% were unaware that nonprescription medicine should not be used in the long term without the advice of a physician or pharmacist. Half of the participants had misperception that all kinds of medication should be stored in the refrigerator.

**Table 2:** Medication knowledge, self-efficacy, and medication literacy by gender.

	Total		Female		Male		T-test
	Mean	SD	Mean	SD	Mean	SD	p value
Knowledge of correct medication use	0.81	0.18	0.83	0.14	0.79	0.21	< .0001
Self-efficacy of correct medication use	4.21	0.83	4.27	0.74	4.16	0.91	< .0001
Medication literacy	4.03	1.25	4.24	1.13	3.83	1.32	< .0001

Notes: SD: Standard deviation; N = 6226

### Self-efficacy of correct medication use

The participants demonstrated high levels of self-efficacy of correct medication use (M = 4.21). Male participants had lower levels of self-efficacy of correct medication use than did female participants (Table 2). Of the self-efficacy items, the participants had the lowest confidence in telling health providers whether or not the medication they were taking contained acetaminophen (M = 3.91).

### Medication literacy

The participants had moderate levels of medication literacy (M = 4.03). Male participants’ medication literacy (M = 3.83) was significantly lower than that of female participants (M = 4.24) (Table 2). In all, 95% of the participants could correctly report the method of medication use on a drug label. However, only 38.3% of the participants could correctly identify the drug classification of the medicine (functional literacy). In addition, 78.1% of the participants could correctly report the expiration date of the medicine (functional literacy), whereas 71.4% could express their personal condition and needs to a pharmacist clearly (interactive literacy). Moreover, only 50.1% could decide whether the medicine would suit their needs (critical literacy), 70.4% correctly responded that a five-year-old girl should not use the same medicine as an adolescent because she is under six years old, even if the two share a symptom (critical literacy). Overall, the interactive and critical levels of medication literacy among the participants surveyed were lower than the functional level.

### Prevalence of SM and inappropriate SM

**Fig 1:**
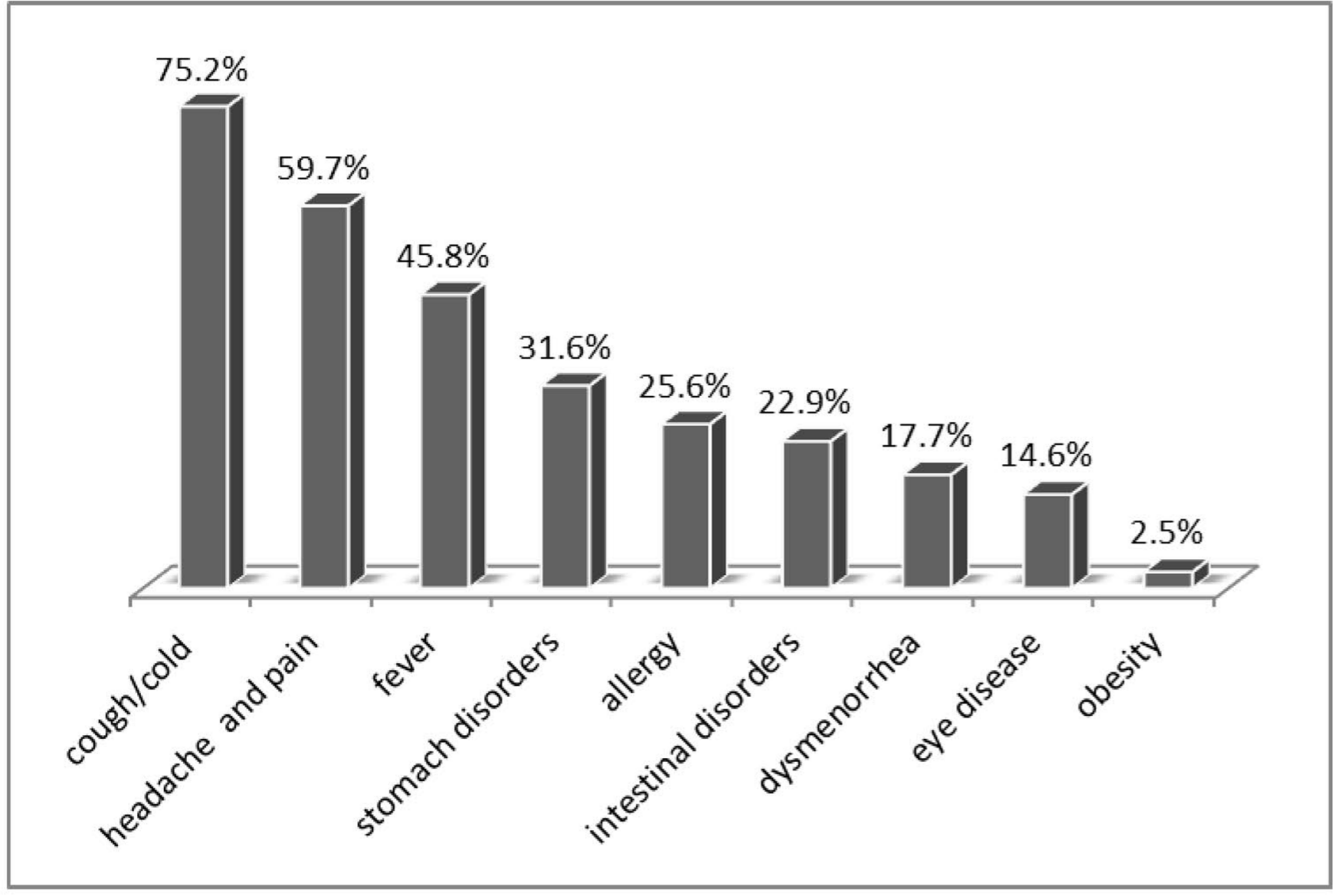
Health complaints among adolescents who self-medicated.

**Fig 2:**
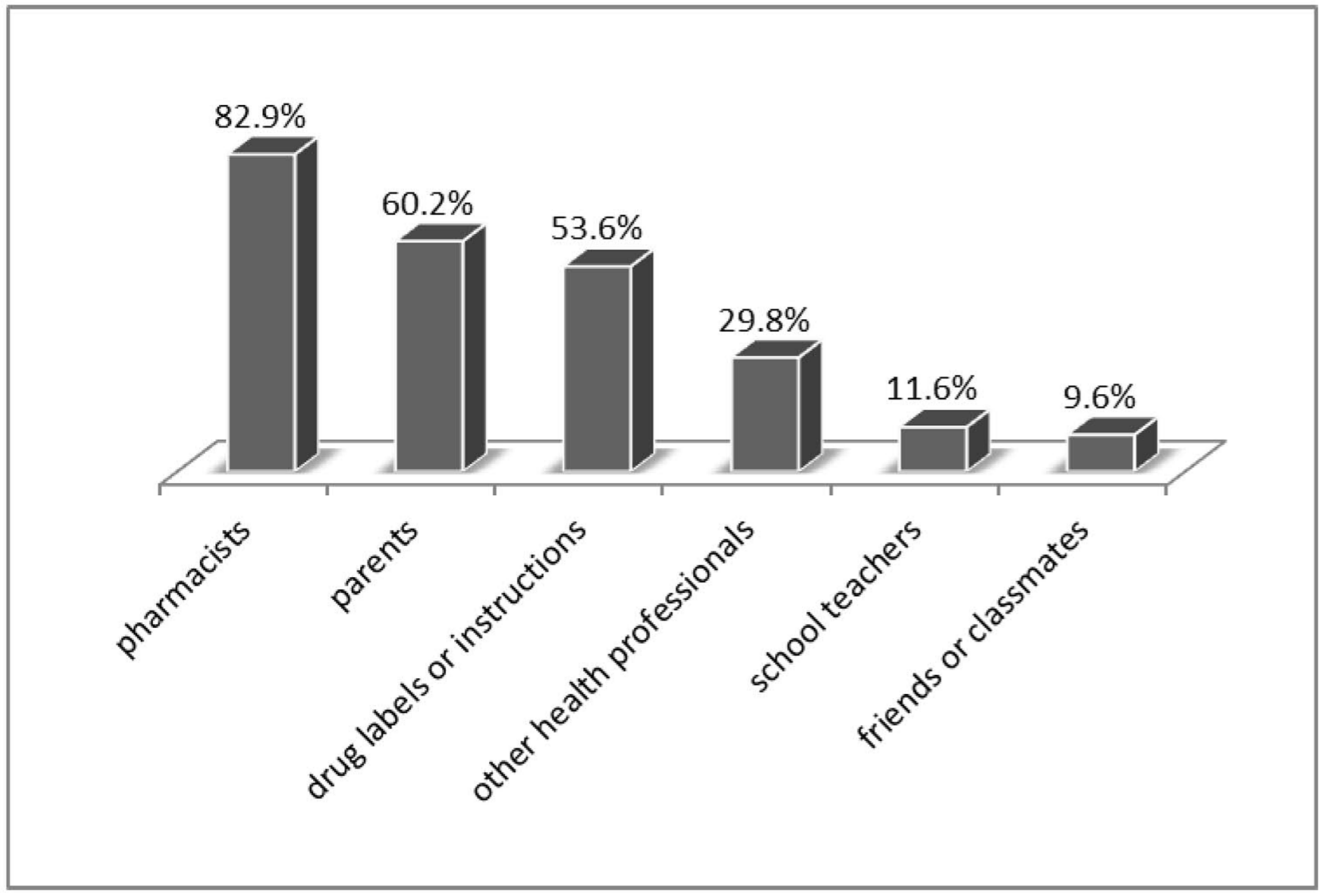
The source of drug information for self-medication.

The prevalence of SM among the participants was 45.7%; it was 45.7% in females and 45.8% in males. The most common health complaints for SM reported by the participants were cough or cold (75.2%), followed by headache (59.7%), fever (45.8%), stomach disorder (31.6%), intestinal disorder (22.9%), allergy (22.6%), eye disease (14.6%), and dysmenorrhea (17.7%, female) (Fig 1). The most frequently used drugs for SM were nonsteroidal anti-inflammatory drugs or pain relievers (prevalence = 31.1%), cold and cough medicines (prevalence = 21.6%), analgesics (prevalence = 19.3%), and antacids (prevalence = 17.3%). Of 2849 self-medicated adolescents, 1892 adolescents (66.4%) self-medicated one drug, 457 (16%) self-medicated two drugs, 291 (10.2%) self-medicated three drugs, and 209 (7.3%) self-medicated four or more drugs in the past year. About one-third of the participants did not retain the packaging and instructions for medications till the time the medicine was completely taken. The main source of information for the drugs used for SM was pharmacists (82.9%), followed by the participant’s parents (60.2%), drug labels or instructions (53.6%), other health professionals besides a physician or pharmacist (29.8%), school teachers (11.6%), and friends or classmates (9.6%) (Fig 2). Of the participants practicing SM, the prevalence of not reading drug labels or instructions before taking medicine was 10.1%, with female participants at 8.3% and male participants at 11.9%; of taking medicines with excessive dosage was 21.6%, with female participants at 13.1% and male participants at 29.7%; and of practicing polypharmacy without being advised by health providers was 30.3%, with female participants at 21.5% and male participants at 38.8%. Male participants engaged in inappropriate SM behaviors more frequently than did female participants (Table 3).

**Table 3:** Prevalence of inappropriate self-medication among adolescents by demographic characteristics and substance use.

	Total SM (n = 2849)	Not reading label (n = 288)		Use of excessive drug dosage (n = 614)		Polypharmacy (n = 864)	
	n	n (%)	p value	n (%)	p value	n (%)	p value
School level			0.3737		<0.0001		<0.0001
Primary	745	84 (11.3)		195 (26.2)		316 (42.4)	
Middle	1058	107 (10.1)		249 (23.5)		331 (31.3)	
High	1046	97 (9.3)		170 (16.2)		217 (20.7)	
Gender			0.0017		<0.0001		<0.0001
Female	1397	116 (8.3)		183 (13.1)		300 (21.5)	
Male	1452	172 (11.8)		431 (29.7)		564 (38.8)	
Household income			0.0505		0.0084		<0.0001
Poor	531	66 (12.4)		137 (25.8)		193 (36.3)	
Middle and above	2318	222 (9.6)		477 (20.6)		671 (28.9)	
Chronic disease			0.9297		0.0341		0.0008
No	2645	267 (10.1)		558 (21.1)		782 (29.6)	
Yes	204	21 (10.3)		56 (27.5)		82 (40.2)	
Family members with a professional health background			0.6099		0.2246		0.0339
No	2640	269 (10.2)		562 (21.3)		787 (29.8)	
Yes	209	19 (9.1)		52 (24.9)		77 (36.8)	
Alcohol use			<0.0001		0.0082		0.0417
No	2547	237 (9.3)		531 (20.9)		757 (29.7)	
Yes	302	51 (16.9)		83 (27.5)		107 (35.4)	
Tobacco use			<0.0003		<0.0001		<0.0001
No	2723	263 (9.7)		558 (20.5)		797 (29.3)	
Yes	126	25 (19.8)		56 (44.4)		67 (53.2)	

Notes: Chi-square tests were conducted; SM: Self-medication

### Factors related to SM

The factors related to SM are shown in Table 4. The results of multiple logistic regression analysis showed that after controlling for gender and school level, students who had family members with a professional health background had better medication knowledge, higher self-efficacy of correct medication use, and higher medication literacy. Further, those who consumed alcohol were more likely to engage in SM.

**Table 4:** Factors related to self-medication.

	Self-medication					
	Simple logistic regression			Multiple logistic regression		
Variable	OR	95% CI	p value	Adj. OR	95% CI	p value
School level						
Middle vs. primary	1.10	0.97–1.25	0.1257	1.09	0.96–1.24	0.1743
High vs. primary	1.11	0.98–1.26	0.1141	1.09	0.96–1.24	0.1850
Gender (male vs. female)	1.00	0.91–1.11	0.9705	0.95	0.86–1.05	0.3503
Household income (Middle and above vs. poor)	0.98	0.87–1.12	0.8052	1.02	0.89–1.16	0.7430
Chronic disease (yes vs. no)	1.22	1.01–1.50	0.0492	1.20	0.97–1.47	0.0872
Family members with a professional health background (yes vs. no)	1.26	1.03–1.54	0.0256	1.25	1.02–1.53	0.0299
Knowledge of correct medication use	1.02	0.78–1.34	0.8808	1.53	1.12–2.09	0.0073
Self-efficacy of correct medication use	0.91	0.85–0.96	0.0010	0.90	0.85–0.96	0.0021
Medication literacy	0.93	0.89–0.96	0.0002	0.93	0.89–0.97	0.0006
Alcohol use (yes vs. no)	1.49	1.25–1.77	< .0001	1.35	1.09–1.66	0.0045
Tobacco use (yes vs. no)	1.63	1.24–2.15	0.0004	1.27	0.91–1.75	0.1558

Notes: Simple and multiple logistic regressions were conducted; OR: Odds ratio; CI: confidence interval; Self-medication model N = 6209 yes n = 2844 no n = 3365

### Factors related to inappropriate SM behaviors

The factors related to inappropriate SM behaviors among the participants practicing SM are shown in Table 5. The results of multiple logistic regression showed that the participants who consumed alcohol and had lower levels of medication knowledge and lower self-efficacy for correct medication use were less likely to read drug labels or instructions before taking medicine. In addition, participants who were male, were tobacco users, had lower levels of medication knowledge, had lower self-efficacy, and had lower medication literacy were more likely to use excessive dosages. Moreover, after controlling for gender, school level, and chronic disease, participants who had lower medication knowledge, lower self-efficacy, and lower medication literacy were more likely to practice polypharmacy without advice from health providers.

**Table 5:** Factors related to inappropriate adolescent self-medication behaviors.

	Not reading label			Use of excessive dosages			Polypharmacy		
	Adj. OR	95% CI	p value	Adj. OR	95% CI	p value	Adj. OR	95% CI	p value
School level			0.7745			0.0020			< .0001
Middle vs. primary	0.96	0.70–1.31	0.7941	1.11	0.87–1.41	0.4050	0.70	0.56–0.86	0.0010
High vs. primary	0.89	0.64–1.24	0.4866	0.73	0.56–0.94	0.0166	0.41	0.32–0.52	< .0001
Gender (male vs. female)	1.25	0.97–1.64	0.0904	2.06	1.68–2.53	< .0001	1.75	1.47–2.10	< .0001
Household income (Middle and above vs. poor)	0.95	0.69–1.29	0.7381	0.99	0.78–1.27	0.978	0.92	0.74–1.15	0.4830
Chronic disease (yes vs. no)	0.91	0.56–1.48	0.7019	1.23	0.85–1.77	0.2699	1.52	1.09–2.12	0.0122
Family members with a professional health background (yes vs. no)	0.85	0.51–1.40	0.5116	1.05	0.73–1.51	0.8094	1.23	0.88–1.70	0.2233
Knowledge of correct medication use	0.42	0.22–0.81	0.0097	0.15	0.09–0.25	< .0001	0.21	0.21–0.35	< .0001
Self-efficacy of correct medication use	0.64	0.56–0.74	< .0001	0.88	0.78–0.99	0.0347	0.85	0.76–0.94	0.0028
Medication literacy	0.97	0.87–1.08	0.5982	0.67	0.62–0.73	< .0001	0.69	0.63–0.74	< .0001
Alcohol use (yes vs. no)	1.84	1.21–2.80	0.0041	0.98	0.67–1.44	0.9353	1.10	0.78–1.55	0.5861
Tobacco use (yes vs. no)	1.03	0.57–1.84	0.9245	1.71	1.02–2.84	0.0401	1.56	0.96–2.54	0.0741

Notes: Multiple logistic regression analysis was conducted; Adj. OR: Adjusted odds ratio; CI: confidence interval; Not reading label model N = 2843 yes n = 287 no n = 2556; Use of excessive dosages model N = 2844, yes n = 611 no = 2233; Polypharmacy model N = 2844 yes n = 862 no n = 1982

## Discussion

This study found that half of the surveyed Taiwanese adolescents had practiced SM in the past year, whereas one-third had engaged in inappropriate SM, such as not reading drug labels or instructions before taking a medicine, taking medicines with excessive dosages, and practicing polypharmacy. In addition, our results indicated that adolescents often did not understand the information on a drug label or instructions—even those that reported reading them before taking the medication. Lower levels of medication knowledge and lower medication literacy were associated with inappropriate SM. Prior studies have indicated that individuals with lower health literacy tend to practice SM frequently [34, 57] and engage in inappropriate SM [34–36]. Since the improvement of medication literacy could enhance an individual’s ability to properly use medication [33], medication literacy enhancement programs should be developed and implemented in schools and communities. Studies have shown that education programs on correct medication use could improve medication knowledge among adolescents [33, 56].

This study found that substance use, such as alcohol consumption and smoking, was associated with SM and inappropriate SM among adolescents. A previous study also found that substance use is associated with SM in adults [58], whereas a prospective study has reported that smoking and alcohol drinking are related to low health literacy and inappropriate medication use [59]. According to the gateway drug theory, an adolescent’s tobacco and alcohol use are associated with later drug abuse [60]. It is critical to implement programs of substance-use prevention combined with correct medication use for adolescents. Future studies could examine the purposes and patterns of self-medicated (OTC or prescription-only) use among adolescents to further examine longitudinal relationships between alcohol or cigarette use and inappropriate SM behaviors.

This study found that gender and school level were associated with SM and inappropriate SM. Prior studies have found that females practiced SM more frequently than males [61–63], but our results indicated that male and female participants have a similar tendency to practice SM. However, participants who were male and had lower school levels were more likely to engage in inappropriate SM behaviors. Since the Taiwanese government does not regulate the age at which medication can be purchased, pharmacists should make a greater effort to help adolescents correctly choose and use medicines, in particular for high-risk groups. In addition, it is critical to continuously implement a correct medication education program combined with life-skill training and media literacy in schools and communities to enhance adolescents’ and parents’ medication literacy and reduce inappropriate SM behaviors.

This study found that pharmacists were the major source of information for SM. Since pharmacists have the professional capacity to provide customized and sound advice on the proper use of medications, governments could provide incentives for pharmacists to play a more proactive role in helping consumers make informed choices, in particular for adolescents and consumers with low health literacy. In addition, adolescents should be educated to enhance their skills and competences in communicating with health providers and critically analyzing drug information and advertisements. Furthermore, consumers should be encouraged to discuss their medication use with pharmacists and other health care providers.

This study had some limitations. First, 9.2% of the sample schools refused to participate in this survey, and potential bias from the selection and refusal to participate must be considered. Second, this study’s results are based on self-reported data and recall bias might have underestimated the prevalence of SM. Third, the three inappropriate behaviors of SM identified in this study might not represent all inappropriate SM behaviors.

## Conclusions

About half of the adolescents surveyed had SM experiences in the past year, whereas one-third practicing SM engaged in inappropriate SM, such as not reading drug labels or instructions before taking medication, taking medication with excessive dosages, and polypharmacy. Multivariate analysis indicated that adolescents with lower medication knowledge, lower self-efficacy, lower medication literacy, and substance use were more likely to engage in inappropriate SM.
